# 西罗莫司成功治疗干扰素及阿糖胞苷治疗失败的Erdheim-Chester病1例

**DOI:** 10.3760/cma.j.issn.0253-2727.2023.04.016

**Published:** 2023-04

**Authors:** 佳雯 戴, 欣欣 曹

**Affiliations:** 中国医学科学院、北京协和医学院北京协和医院血液内科，北京 100730 Department of Hematology, Peking Union Medical College Hospital, Chinese Academy of Medical Sciences and Peking Union Medical College, Beijing 100730, China

患者，女，35岁，因“干咳1年，活动后喘憋5个月”于2019年12月就诊于我院。患者2018年10月无诱因出现干咳，2019年7月起干咳加重，逐渐出现活动后呼吸困难。患者就诊于外院，B超提示心包积液、双侧大量胸腔积液，取胸腔穿刺引流液行病原学、结核DNA及瘤细胞检测均未见异常。筛查抗核抗体谱、肿瘤标志物均为阴性。行PET/CT：心包稍增厚，代谢轻度增高，SUVmax 3.5，双侧肱骨头及双侧肱骨上段、骶椎、双侧髂骨、右侧坐骨及双侧股骨上段见多发结节状异常代谢增高灶，SUVmax 13.1，双附件各见一结节状代谢浓聚，SUVmax 5.6，腹膜后、肠系膜间隙多发淋巴结肿大，SUVmax 6.7。完善全身骨显像：双侧胫骨中上段及两侧股骨下段、双侧腓骨中上段硬化性改变，伴颅骨、双肩关节、双侧肱骨中上段、双侧骶髂关节、双侧髋关节、双侧股骨大转子骨质骨显像代谢对称性活跃。头增强MRI：双侧眶内、视神经及眼肌信号异常，右侧丘脑异常信号，水肿可能。2019年10月行腹腔镜活检术，术中见腹盆腔内大量积液，腹壁、网膜表面大量粟粒样结节。取病理送检：（我院病理会诊）脂肪组织内见组织细胞、树突状细胞及肌纤维母细胞弥漫性增生，伴少量淋巴细胞、浆细胞浸润，未见细胞异型性，考虑弥漫性组织细胞、树突状细胞及肌纤维母细胞增生性病变。免疫组化：CD163（+），CD68（+），S-100（+），CD1a（−）。2019年11月起予甲泼尼龙40 mg/d治疗，激素治疗近1个月后复查浆膜腔积液较前无减少。患者为进一步诊治于我院血液科住院治疗。

查体：突眼，颈静脉显露，双侧甲状腺Ⅱ度肿大、质韧，双下肢轻度对称可凹性水肿。实验室检查：生化常规：肝肾功能、乳酸脱氢酶正常，白蛋白26 g/L；炎症指标：红细胞沉降率 11mm/1 h，超敏C反应蛋白 81.61 mg/L，白细胞介素-6 107.0 pg/ml，肿瘤坏死因子-α 14.0 pg/ml；甲状腺功能：T3 0.39 ng/ml，T4 37.0 µg/L，游离T3 1.66 pg/ml，游离T4 9.6 ng/L，促甲状腺素 87.629 µIU/ml。性激素、血及尿渗透压正常。血清蛋白电泳、免疫固定电泳、抗核抗体谱、IgG亚类、乙肝病毒DNA均正常。胸腔积液及心包积液均为渗出液。病理组织二代测序：未检测到BRAF^V600E^突变及其他MAPK通路突变。胸腹盆增强CT+CTA：纵隔内弥漫软组织密度，主动脉及分支受包绕；双肺近心包及肺门区周围软组织团片影；腹主动脉下段管壁周围软组织增厚；双侧胸膜、心包、腹膜、肠系膜不规则增厚伴多发软组织密度结节；肾窦区软组织增厚。结合影像学检查及病理结果，考虑Erdheim-Chester病（ECD）诊断明确，骨、心包、腹膜、血管、肠系膜、双肾、眶后及甲状腺受累。2019年12月8日起开始干扰素治疗，具体为：重组人干扰素α2a注射液900万国际单位皮下注射，隔日一次。患者甲状腺功能减低，予左甲状腺素钠片替代治疗。2020年6月复查PET/CT提示原代谢增高病灶均较前好转，继续干扰素治疗。2020年12月再次复查PET/CT：腹膜及心包病变较前加重，腹水及心包积液较前增多，考虑病情进展。2020年12月29日起改用中剂量阿糖胞苷化疗，具体为：阿糖胞苷1 g/m^2^，2次/d，共3 d。1个疗程后患者腹腔积液进一步增多，考虑治疗效果不满意，第2个疗程起联合干扰素900万国际单位，隔日一次。4个疗程后于2021年5月复查PET/CT：心包积液及腹腔积液较前增多、皮下新发病灶，考虑病情再次进展。2021年5月20日起调整治疗为雷帕霉素（西罗莫司）1 mg/d，联合泼尼松0.75 mg·kg^−1^·d^−1^治疗。第2个月激素减至0.5 mg·kg^−1^·d^−1^，第3至4个月减至0.25 mg·kg^−1^·d^−1^，第5至6个月减至0.125 mg·kg^−1^·d^−1^，半年后逐渐减量至5 mg/d，长期维持。用药后患者浆膜腔积液未进一步增加。治疗6个月后复查PET/CT：上述所有病灶SUV值均较前明显降低，考虑治疗有效。目前患者仍继续接受雷帕霉素治疗中。

